# General-purpose topology-aware embedding of tumor phylogenetic trees with graph neural networks

**DOI:** 10.1093/bioadv/vbag016

**Published:** 2026-04-26

**Authors:** Paolo Bresolin, Fabio Vandin

**Affiliations:** Department of Information Engineering, University of Padova, Padova 35131, Italy; Department of Information Engineering, University of Padova, Padova 35131, Italy

## Abstract

**Motivation:**

Phylogenetic trees are tree-like data structures commonly adopted to mathematically represent cancer clonal evolution. The information encoded by phylogenetic trees is important for clinical outcomes, but the automatic extraction of such information is still hard, also due to the fact that working directly with tree-like data structures is complex. This is especially true for machine learning tasks, where models are usually designed for vector data.

**Results:**

We introduce CPhyT-GNN, a novel Deep Learning method to compute unsupervised embeddings of phylogenetic trees. The embeddings learnt by CPhyT-GNN are vectors that can be used for a variety of machine learning tasks. CPhyT-GNN is based on Graph Neural Networks, which allow to obtain representations that combine the information provided by the alterations present in the tumor and the topological information provided by the corresponding phylogenetic tree. Experiments with cancer data show that the embeddings learnt by our model are general-purpose and can be applied to different tasks, with results that improve the state-of-the-art.

**Availability and implementation:**

Data and code are available at the following link: https://github.com/VandinLab/CPhyT-GNN.

## 1 Introduction

Cancer is a widespread disease characterized by the accumulation of somatic alterations by different subpopulations of cells (Nowell 1976). This process is usually known as *cancer clonal evolution*, where a *clone* is a subpopulation of cells that share the same set of somatic alterations. One of the features of cancer is its high heterogeneity (Heppner *et al.* 1981), present at different levels in tumors. More specifically, *inter-tumor* heterogeneity refers to the observation that tumors affecting patients of the same cancer type may have considerably different somatic alterations. Furthermore, the coexistence of multiple clones within the same tumor reveals an additional level of *intra-tumor* heterogeneity, which has impact on clinical outcomes (Marusyk and Polyak 2010, Ramón y Cajal *et al.* 2020).

Nevertheless, patterns of evolution common to different tumors can still be found (Hanahan and Weinberg 2000) and exploited to gain insights from the increasing amount of data accessible nowadays thanks to DNA bulk sequencing technologies (Gerlinger *et al.* 2012, Williams *et al.* 2018) and modern single-cell sequencing techniques (Morita *et al.* 2020, Lei *et al.* 2021, Li and Wang 2021). Starting from sequencing data, several methods have been developed to infer tumors’ phylogenetic trees (Deshwar *et al.* 2015, El-Kebir *et al.* 2016, Jahn *et al.* 2016, Satas and Raphael 2017, Dang *et al.* 2017, Baghaarabani *et al.* 2021), tree-like data structures representing the evolutionary relations among the clones in a tumor. This flourishing research paved the way to methods that extract common evolutionary patterns from phylogenetic trees (Vandin *et al.* 2011, Caravagna *et al.* 2018, Khakabimamaghani *et al.* 2019, Christensen *et al.* 2020, Pellegrina and Vandin 2022, Govek *et al.* 2022, Luo *et al.* 2023, Ivanovic and El-Kebir 2023).

While the use of phylogenetic trees as input to cancer progression models has been extensively studied, their integration for solving specific tasks, such as the prediction of clinical outcomes, is still limited. A direction still unexplored is to learn *embeddings*, i.e. general-purpose vector representations, from phylogenetic trees. To the best of our knowledge, the only model for phylogenetic trees embedding is Oncotree2vec (Baciu-Drăgan and Beerenwinkel 2024), based on a Neural Network (NN) language model. The main drawback of Oncotree2vec is that it cannot be applied to embed phylogenetic trees not used for training, making it unsuitable for prediction tasks that involve unseen data. Moreover, Oncotree2vec is based on handcrafted features and requires the user to specify a weight defining the importance of each type of feature before training the model. Another limitation is that it can be applied only to mutation trees, i.e. phylogenetic trees where each node is labeled by exactly one alteration, while several tumor trees methods produce in output trees with multiple alterations for each node (Deshwar *et al.* 2015, El-Kebir *et al.* 2016, Satas and Raphael 2017, Dang *et al.* 2017, Baghaarabani *et al.* 2021), in part due to the uncertainty in the order of alterations inferred from sequencing data. In addition, Oncotree2vec has been designed mostly for clustering, especially to distinguish patients affected by different cancer types rather than patients in the same cancer cohort.

Deep Learning (DL), that has shown impressive results in many fields in the last few years, is of particular interest for learning embeddings from tumor trees, thanks also to the increasing number of available large datasets of phylogenetic trees for tumors. In particular, since a phylogenetic tree is a special case of graph, the adoption of models based on Graph Neural Networks (GNNs) (Kipf and Welling 2016, Hamilton *et al.* 2017) appears a natural choice. Indeed, GNNs can be used to extract information from both the variants appearing in a phylogenetic tree and its topology. At the time of writing, no method based on GNNs to embed phylogenetic trees exists in the literature.

In this work, we present Cancer Phylogenetic Trees GNN (CPhyT-GNN), the first DL method to extract vector representations from phylogenetic trees in an unsupervised fashion. The model is based on a GNN that takes as input a phylogenetic tree and outputs its embedding in $R^{q},\;q\geq 1$. We train the model following an unsupervised approach, so to obtain vector representations that can be used in different downstream tasks. Moreover, our method is designed to work following a very general definition of phylogenetic tree that allows for alteration clusters as well as missing or unknown alterations. We also design a node encoding strategy specific for nodes in tumor phylogenetic trees. Note that, differently from Oncotree2vec, our model is not based on the design of handcrafted structural features, thanks to the GNN that automatically captures complex topological relations. We show the effectiveness of our model on both synthetic and real data. In particular, on real data we show the wide applicability of the embeddings computed by our model by using them in two different tasks: cancer progression modeling and survival analysis. The results show that our model significantly improves the performances of state-of-the-art methods for both the cancer progression modeling task and the prediction of survival times.

## 2 Methods

### 2.1 Problem formulation

The input is a set of phylogenetic trees $D=\{T_{1},\ldots ,T_{n}\}$, where $T_{i}\in D$ is the phylogenetic tree associated to patient i∈[n]={1,…,n}. A *phylogenetic tree* is a rooted directed tree T=(V,E), where *V* is a set of nodes and *E* is a set of directed edges. Every node v∈V is labeled by a set of alterations $L_{v}$, where an alteration can be a Single Nucleotide Variant (SNV), a Copy Number Aberration (CNA) or any other type of somatic alteration, including the loss of previously acquired alterations. A directed edge (u,v)∈E between two nodes u,v∈V points out that the alteration set $L_{v}$ was acquired after the alteration set $L_{u}$ in the tumor evolutionary process specified by *T*. The root r∈V represents the germline, the subpopulation of cells without any somatic alteration acquired, labeled by the empty set $L_{r}=\emptyset$.

Given a phylogenetic tree T=(V,E) with root r∈V and a node v∈V, we define as $\Pi_{r,v}\subseteq V$ the set of all nodes appearing in the directed path from *r* to *v* in *T*. For each node v∈V, we define the *clone* induced by *v* as the set $c(v)=\underset{u\in \Pi_{r,v}}{\cup }L_{u}$ containing all alterations that label the nodes in the path $\Pi_{r,v}$. A clone c(v) induced by a node v∈V represents a subpopulation of cells present in the tumor described by *T* such that all cells are characterized by the same set c(v) of alterations.

Differently from the vast majority of works regarding cancer clonal evolution, we do not require the *infinite sites assumption*, stating that once an alteration is acquired in a tumor, it is never lost and cannot be acquired anymore. This assumption has been shown (Kuipers *et al.* 2017) to be frequently violated in practice. As a consequence, we allow multiple nodes in the same phylogenetic tree to be labeled by the same alteration. Furthermore, we admit $L_{v}=\emptyset$ even for nodes v≠r, allowing for analyses with incomplete input phylogenetic trees.

Let M be the set of all possible tumor alterations appearing in tumors belonging to the considered cancer type, possibly not all present in the input dataset *D*. Define as [m]={1,…,m}⊆M the set of alterations actually appearing in *D*. Let T be the set of all possible phylogenetic trees with nodes labeled by alterations in M. Our objective is to find a mapping $f_{\theta }:T\to R^{q},\;q\geq 1$, where θ is the set of learnable parameters that specify the function $f_{\theta }$ modelled by our method, such that $f_{\theta }(T)$ preserves the evolutionary information stored in T∈T. We define as *embedding* of a phylogenetic tree *T* its vector representation $f_{\theta }(T)\in R^{q}$.

Note that we do not restrict to the alteration set [*m*], but our goal is more general, because we require our mapping $f_{\theta }$ to be capable of embedding phylogenetic trees T∉D with possible unseen alterations. In the ML framework, the problem can be seen as an *unsupervised representation learning* task, where the goal is to train a model that learns general vector representations that can be directly used in different applications. More precisely, we want to train a DL model on *D* so to obtain embeddings that preserve the evolutionary information originally contained in the corresponding phylogenetic trees. In the next section, we show how this can be obtained in an unsupervised way by choosing an appropriate loss function to be minimized during training.

### 2.2 Loss function

Let D be the unknown distribution over T and denote by T∼D a phylogenetic tree sampled from T according to D. Let $d:T\times T\to R^{+}$ be a phylogenetic tree distance function, quantifying the difference between two input phylogenetic trees. Given a model $f_{\theta }$ with parameters θ, we define the loss for $T_{i},T_{j}∼D$ as:


(1) $$L(T_{i},T_{j}|\theta )=\left|d(T_{i},T_{j})-\left\|f_{\theta }(T_{i})-f_{\theta }(T_{j})\right\|\right|.$$

That is, the loss quantifies the difference between the distance $d(T_{i},T_{j})$ of $T_{i}$ and $T_{j}$ and the distance of the corresponding embeddings $f_{\theta }(T_{i})$ and $f_{\theta }(T_{j})$. Therefore, the expectation of the loss over the distribution D is $L(D|\theta )=E_{T_{i},T_{j}∼D}[L(T_{i},T_{j}|\theta )]$ and our goal is to find the set of model parameters $\theta^{⋆}$ such that $\theta^{⋆}=\arg\min_{\theta }\;L(D|\theta )$. Since D is unknown, we define the loss function to be optimized by our model during training as the average loss over all pairs of phylogenetic trees in $D=\{T_{i}∼D:i\in [n]\}$:


(2) $$L(D|\theta )=\frac{1}{\left(\begin{array}{c} \left|D\right|\\ 2 \end{array}\right)}\sum_{\{T_{i},T_{j}\}\subseteq D}L(T_{i},T_{j}|\theta ).$$

In the literature, there are several distance measures used to compare phylogenetic trees that represent tumor evolution (DiNardo *et al.* 2020, Govek *et al.* 2022, Vasei *et al.* 2024). Our model potentially works with any distance function $d:T\times T\to R^{+}$. In particular, we decide to extend the Ancestor-Descendant Distance (Govek *et al.* 2022) to our notion of phylogenetic tree, as explained in Supplementary Material A.1, available as supplementary data at *Bioinformatics Advances* online.

### 2.3 Phylogenetic tree encoding

A crucial preliminary step to consider when adopting a GNN is the choice of an effective encoding for the nodes of the input graphs. Since we want our model to extract embeddings also for unseen phylogenetic trees, we need an encoding that works for any T∈T, with alterations in M. However, a first problem is that M is only partially known. Indeed, the available data is a finite set of phylogenetic trees $D=\{T_{i}\in T:i\in [n]\}\subset T$ with set of alterations appearing in *D* that is [m]⊆M. Therefore, it may happen that, after learning $f_{\theta }$ from a set of tumor phylogenetic trees, we need to encode a phylogenetic tree T∈T∖D with unknown alterations in M∖[m]. Secondly, we need to properly encode nodes *v* with no alteration label $L_{v}=\emptyset$. Moreover, the root node r∈V of every phylogenetic tree has $L_{r}=\emptyset$, but has a different meaning with respect to an empty node, so it is necessary to distinguish it from the other nodes in *T*.

We compute an intermediate encoding $\lambda_{v}\in Z^{m+2}$ for every node v∈V of the input phylogenetic tree T=(V,E), which is based on the alteration set $L_{v}\subset M\cup \{0\}$ that labels *v*. In particular, each component i∈[m] of $\lambda_{v}$ is set to $\lambda_{v}[i]=1$ if $i\in L_{v}$ and $\lambda_{v}[i]=0$ otherwise. Component $\lambda_{v}[0]$, instead, is used to encode the root node *r*, which is represented by $\lambda_{r}={\left[1,0,\ldots ,0\right]}^{⊺}$, while $\lambda_{v}[0]=0$ for every other node v∈V∖{r}. Finally, $\lambda_{v}[m+1]$ is used to store information about unknown alterations in M∖[m] that may appear in $L_{v}$. More precisely, $\lambda_{v}[m+1]$ is incremented by 1 every time an unknown alteration is found in $L_{v}$ and therefore indicates the number of unknown alterations that label *v*.

The final encoding $x_{v}\in Z^{m+2}$ is computed for each node v∈V by summing up the alteration-based encodings $\lambda_{u}$ of the nodes *u* in the path $\Pi_{r,v}$. This step is added to enrich the node encodings. Indeed, $x_{v}$ can be interpreted as a clone-based encoding for v∈V since the clone induced by *v* is defined as $c(v)=\underset{u\in \Pi_{r,v}}{\cup }L_{u}$. Formally, the final encoding $x_{v}\in Z^{m+2}$ for each node v∈V can be easily computed as $x_{v}=\sum_{u\in \Pi_{r,v}}\lambda_{u}$. Note that a limitation of such an encoding is that two phylogenetic trees with the same topology, same labeling of the nodes and same nodes affected by the same number of different unknown alterations will be mapped to the same embedding. However, this situation is unlikely in many scenarios, and the encoding still provides more information than filtering unknown alterations.

### 2.4 Model architecture

Our goal is to have a model that does not only consider the presence or absence of alterations in a tumor, but also the evolutionary relations among them. Therefore, we propose CPhyT-GNN, a model based on a GNN that takes as input a phylogenetic tree and outputs the corresponding embedding. Given as input a dataset of phylogenetic trees $D=\{T_{1},\ldots ,T_{n}\}$, the training process is unsupervised and the objective is to have embeddings that can potentially be used for several distinct ML tasks.

**Figure 1:**
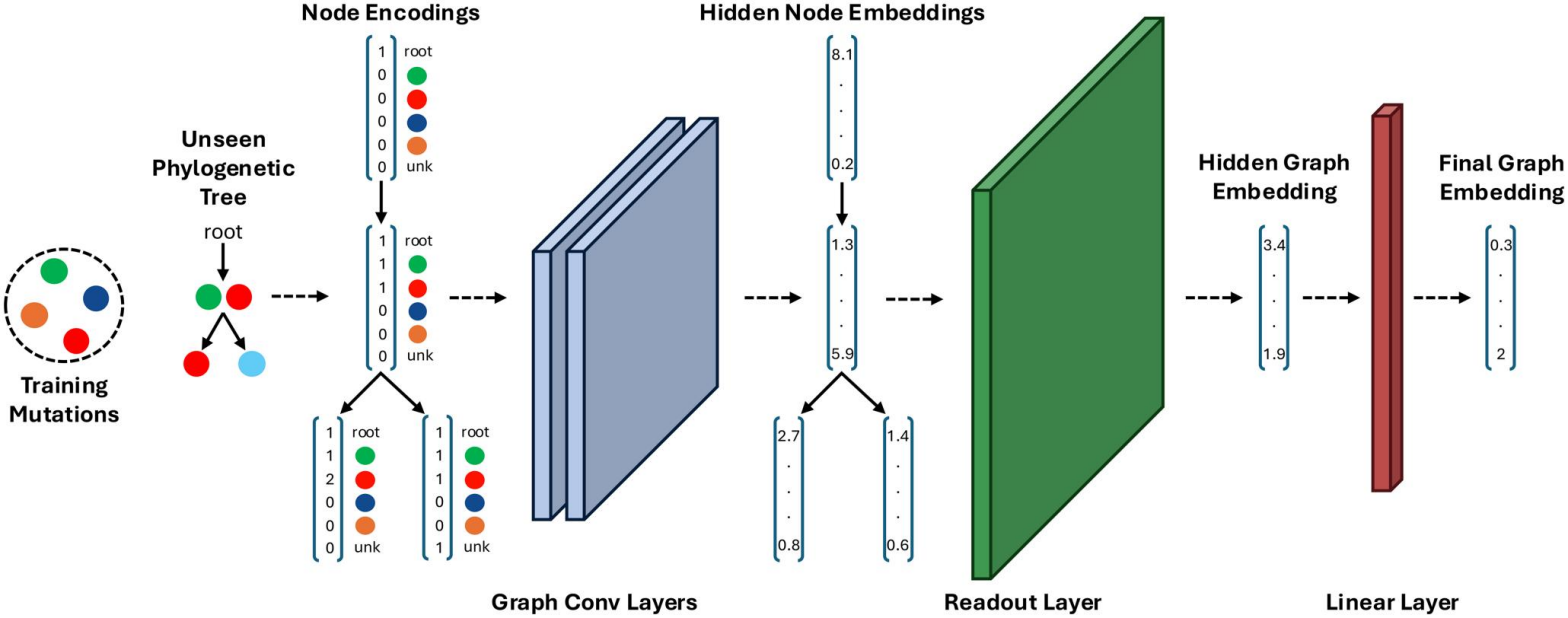
Model architecture. In this example, the embedding of an unseen phylogenetic tree with four alterations is computed using our model, previously trained on a training set with alterations [m]={1,2,3,4}, as reported in the figure. First, the nodes of the input phylogenetic tree are encoded and fed to a GNN. More specifically, two graph convolutional layers are applied so to extract node-level representations, aggregated afterwards into a tree-level hidden encoding via a readout layer that computes the average of the node embeddings. Finally, a fully connected linear layer is applied to map it to the final embedding space $R^{q}$.

The complete model architecture is summarized in Fig. 1. Each node v∈V of the input phylogenetic tree T=(V,E) is encoded as $x_{v}\in Z^{m+2}$ as explained in the previous section. Then, two Graph Convolutional Layers (GCLs), each followed by the application of an activation function σ, are used to learn representations for nodes. Defined as $g_{\theta }^{\left(1\right)}$ and $g_{\theta }^{\left(2\right)}$ the application of the two GCLs, the hidden representations $h_{v}^{\left(1\right)}=\sigma (g_{\theta }^{\left(1\right)}(x_{v}))$ and $h_{v}^{\left(2\right)}=\sigma (g_{\theta }^{\left(2\right)}(h_{v}^{\left(1\right)}))$ are computed ∀v∈V. Thanks to the GCLs, the computed $h_{v}^{\left(2\right)}$ combines the information originally stored in *v* with the information in the other nodes of *T*, based on the topology of *T*.

Since we are interested in embedding the entire phylogenetic tree *T*, the node-level representations $h_{v}^{\left(2\right)}$ are aggregated into a single tree-level embedding $h_{T}\in R^{p}$ using an Average Pooling Layer (APL) that computes $h_{T}=\frac{1}{\left|V\right|}\sum_{v\in V}h_{v}^{\left(2\right)}$. Finally, a Fully Connected Layer (FCL) is applied to refine $h_{T}$ and map it to the final embedding $z_{T}=Wh_{T}+b\in R^{q}$, where $W\in R^{q\times p}$ and $b\in R^{q}$ are, respectively, the weights and the bias of the FCL.

Batch normalization and dropout layers are also added to prevent the model from overfitting the training set. More specifically, each GCL is followed by a batch normalization layer before ReLU activation is applied while dropout is inserted before the second GCL and ahead of the final FCL.

## 3 Results

In this section, we present the experimental evaluation of our model. In particular, we use simulated data to assess whether our model can distinguish phylogenetic trees not only based on the alterations, but also considering the topology of the trees. Next, we show using real data that the unsupervised embeddings computed by our model can be used in different downstream tasks: cancer progression modeling and survival time analysis.

### 3.1 Simulations

The main objective is to evaluate whether our model is capable of distinguishing phylogenetic trees with different topological structures. To this end, we performed five simulations, considering different synthetic datasets. In particular, in simulations I and II we generate phylogenetic trees with random alterations, but different topologies. In simulation III we create unlabeled phylogenetic trees that differ both in terms of sizes and topological structures. Simulations IV and V, instead, take into account phylogenetic trees all with the same topology, but different relations between two fixed alterations that are common and discriminative in real tumor phylogenetic trees. All the details regarding simulations are in Supplementary Material B, available as supplementary data at *Bioinformatics Advances* online.

In each simulation, we generate clusters of phylogenetic trees that share some property and we train our model on the whole dataset, extracting an embedding for each tree in an unsupervised way. Afterwards, the phylogenetic trees are clustered, giving as input their embeddings to the Lloyd’s algorithm (Lloyd 1982). The produced clustering is compared to the ground truth using the Rand Index (RI).

**Figure 2:**
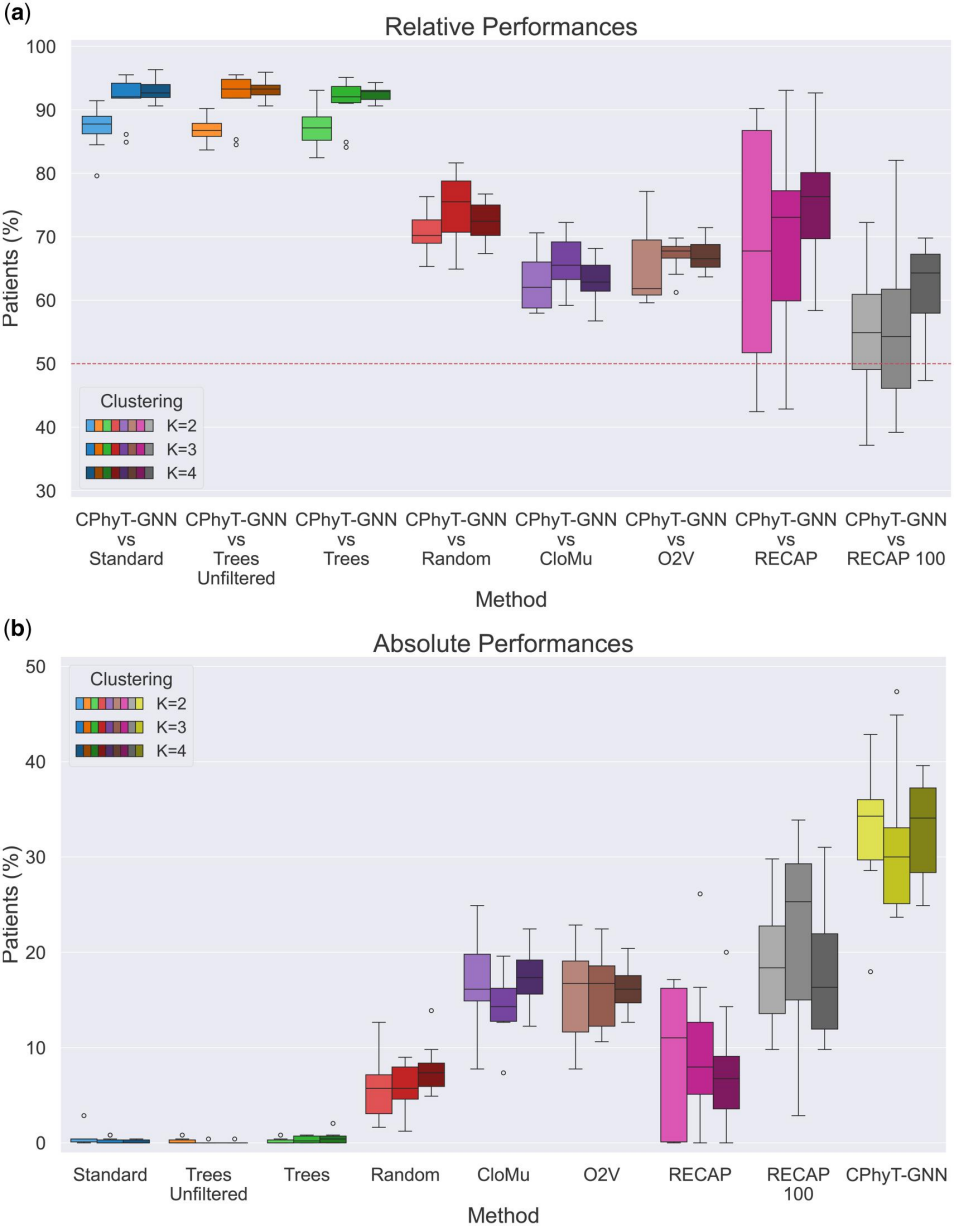
Comparison of different methods on the breast cancer dataset. *RECAP* 100 refers to *RECAP* with F=100; *RECAP* is the application of RECAP with F=0; *Trees Unfiltered* is the method that directly clusters trees using the Ancestor-Descendant distance; *Tree* is *Trees Unfiltered* applied after having filtered out some outlier trees; *O2V* stands for Oncotree2vec; *Standard* is the standard application of CloMu without EL; *CloMu* refers to the CloMu-based baseline. Each box plot corresponds to 10 experiment repetitions. Brightness encodes different values of K∈{2,3,4}. The line in each box plot represents the median value and the empty circles are outliers. (a) Percentage of test patients whom our model assigns a larger score than each baseline. The dashed line highlights the value 50%. (b) Percentage of test patients whom each method assigns a score larger than all other models.

**Figure 3:**
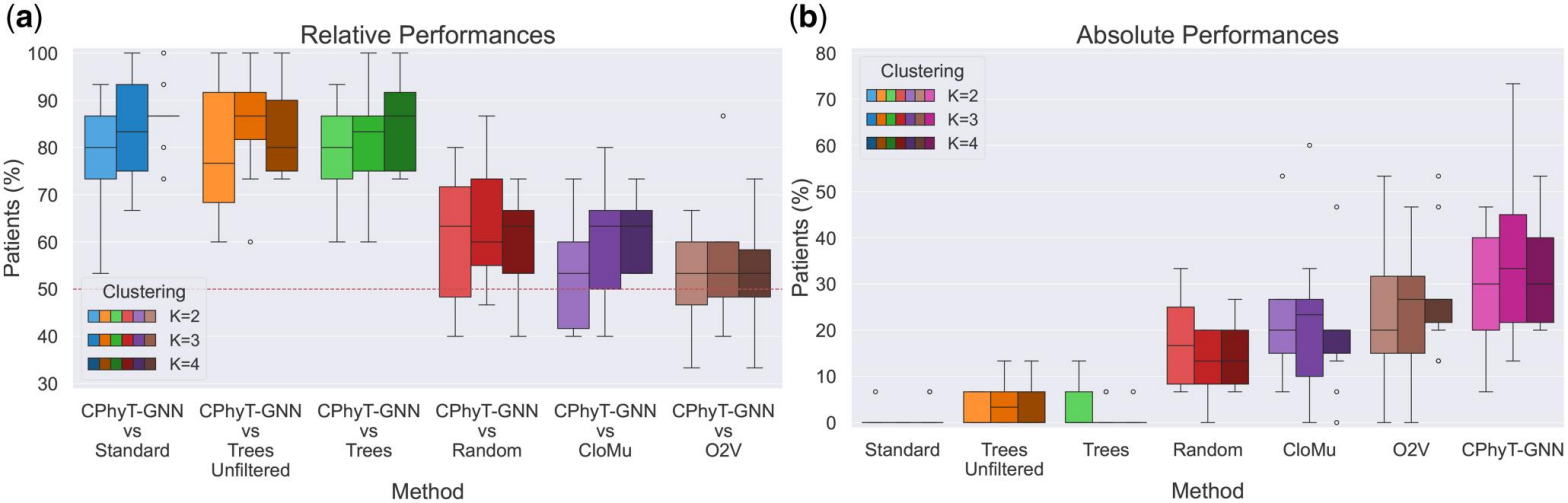
Comparison of different methods on the AML dataset. All alterations are considered (F=0). Each box plot is built considering 10 experiment repetitions. Brightness highlights different values of K∈{2,3,4}. The median is reported for each box plot as a line, circles represent outliers. (a) Percentage of test patients whom our model assigns a larger score with respect to a specific method. The dashed line highlights the value 50%. (b) Percentage of test patients whom the corresponding method assigns a score larger than all other methods.

**Figure 4:**
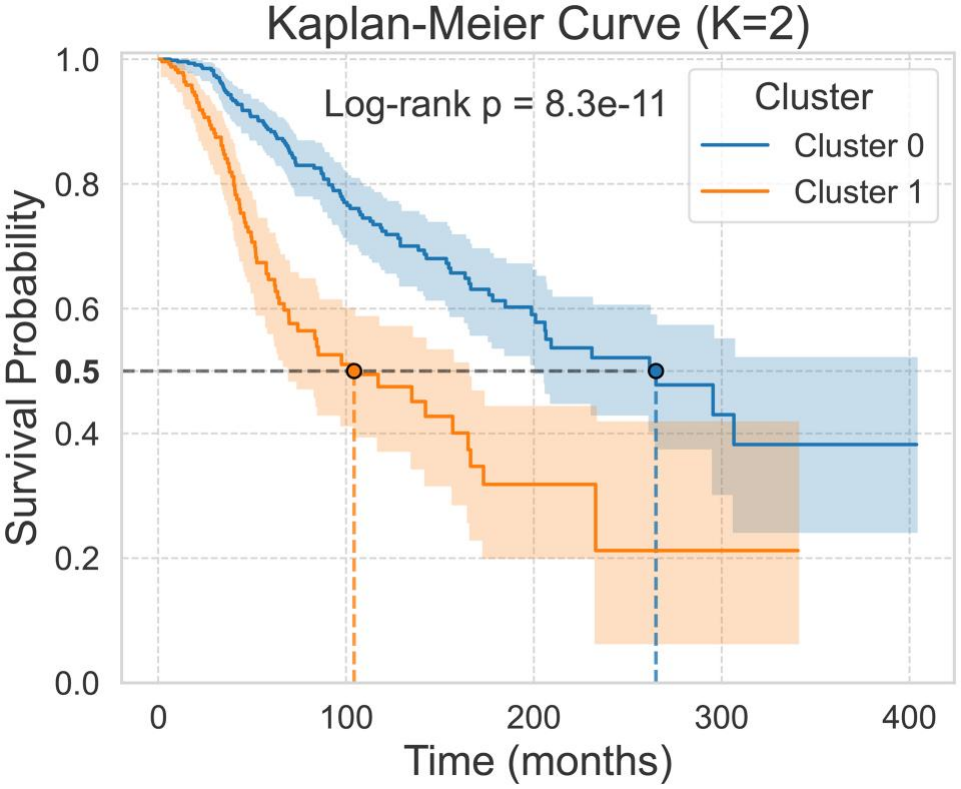
Survival time distributions for the K=2 clusters detected by CPhyT-GNN on the breast cancer dataset. The 95% confidence intervals for the estimates are shown around each curve. The *P* value from the log-rank test is reported.

Our model reaches very high performances in simulations I and II, even when the random noise increases, as shown in Figs 2 and 3, available as supplementary data at *Bioinformatics Advances* online. Our model has high performances in simulation III as well, with an RI always around 0.9, as reported in Fig. 4, available as supplementary data at *Bioinformatics Advances* online. Simulation IV is more complex and, indeed, the RI of our method is more variable, but still high. Finally, our model exhibits perfect results when applied to simulation V, as displayed by Fig. 8, available as supplementary data at *Bioinformatics Advances* online. These results clearly show that our method is capable of distinguishing different phylogenetic trees even when they cannot be distinguished by alterations alone, thanks to the topological structures revealed by the GNN at the basis of our method.

### 3.2 Cancer progression modeling

The objective of this task is to build a model of the clonal evolution of tumors for a given cancer type. For our experiments, we consider CloMu (Ivanovic and El-Kebir 2023), a state-of-the-art method based on Deep Reinforcement Learning (DRL) for modeling cancer progression. CloMu takes as input a dataset of phylogenetic trees representing tumors of the same cancer type. At inference time, CloMu assigns to an unseen tree a score that is the probability of generating such a phylogenetic tree according to the parameters learnt in the training phase. Recently, the DRL model of CloMu has been extended for evolution-aware copy number calling (Ivanovic and El-Kebir 2025).

One of the main problems when modeling cancer clonal evolution is inter-tumor heterogeneity. Indeed, it is very difficult to generalize the behavior of tumors belonging to a given cancer type and, in general, not that useful. Therefore, leveraging on this well-known property of tumors, we propose an Ensemble Learning (EL) method, based on phylogenetic embeddings extracted by our model, that enhances the performances of CloMu.

Given a dataset $D=\{T_{1},\ldots ,T_{n}\}$ of phylogenetic trees, instead of training CloMu directly on *D*, we partition it into K≥2 disjoint subsets $C_{K}=\{C_{1},\ldots ,C_{K}\}$ and train a different instance $I_{i}$ of CloMu independently on each cluster $C_{i},\;i\in [K]$. When an unseen phylogenetic tree T∉D has to be evaluated, it is given as input to all the *K* trained instances, each of which outputs a score $s_{i}=I_{i}(T)$. Then, the maximum of the scores $s_{M}=\max\{s_{i}:i\in [K]\}$ is assigned to *T*. The idea is to perform patients subtyping based on inter-tumor heterogeneity, exploiting the information encoded by the corresponding phylogenetic trees. For this purpose, clusters have to be constructed such that patients with similar tumor evolutions are grouped together and patients with dissimilar tumor evolutions are in different clusters. This is where our model comes into play: we train it on *D* so to extract embeddings $f_{\theta }(T_{i})\;\forall T_{i}\in D$ that are then clustered into *K* groups. In principle, any clustering algorithm that works with vectors can be chosen and we opted for one of the most used, the Lloyd’s algorithm (Lloyd, 1982) in the *K*-means framework.

To assess the quality of the embeddings produced by our model, we use the same EL approach with different baselines for clustering the input phylogenetic trees. The first one is a random clustering of *D*, with clusters’ dimensions that match those returned by our model. Second, we consider the CloMu-based baseline detailed in Supplementary Material C.1, available as supplementary data at *Bioinformatics Advances* online, where we start from the random clustering computed with the baseline above and we refine it by training a CloMu model on each cluster and re-assigning trees based on the probabilities from the trained models. Furthermore, we consider the clustering computed by applying hierarchical clustering directly to the input phylogenetic trees, using the Ancestor-Descendant distance as measure for clustering, since the same measure is used to train CPhyT-GNN. We also compare with Oncotree2vec (Baciu-Drăgan and Beerenwinkel 2024), which is state-of-the-art for embedding a dataset of phylogenetic trees. In addition, we consider RECAP (Christensen *et al.* 2020), a state-of-the-art method to cluster patients from an input set of phylogenetic trees. The standard application of CloMu trained on the whole training set *D* is included as additional baseline to understand whether the EL strategy that we propose is effective. Note that, while we expect the standard application of CloMu to have worse performances than the other methods, since it is the only model that exploits a single CloMu instance to compute probabilities rather than taking the maximum of K>1 probabilities, this depends on the quality of the clustering. In fact, if the similarities of the trees in each cluster are the same as the similarities in the overall set of trees, the best of the *K* CloMu models will have a similar performance as the CloMu model trained using all trees.

The two real world datasets that we consider for this task (Razavi *et al.* 2018, Morita *et al.* 2020) have patients with multiple plausible phylogenetic trees. CloMu and RECAP were designed to deal with uncertainty in multiple phylogenetic trees per patient; in Supplementary Material C.2, available as supplementary data at *Bioinformatics Advances* online, we explain how we deal with this situation and adapt the other methods. Moreover, in Supplementary Material C.4, available as supplementary data at *Bioinformatics Advances* online, we provide the details of a filtering procedure that we plug-in to Oncotree2vec, CPhyT-GNN and the tree distance baseline with the objective of improving clustering performances.

#### 3.2.1 Breast cancer

The dataset consists of n=1315 patients affected by breast cancer (Razavi *et al.* 2018). Phylogenetic trees were reconstructed by the authors of CloMu (Ivanovic and El-Kebir 2023) by applying SPRUCE (El-Kebir *et al.* 2016) to bulk sequencing data with a gene panel, where only SNVs were considered. We performed exactly the same pre-processing steps described in Ivanovic and El-Kebir (2023), removing phylogenetic trees with more than nine edges, ending up with a total of n=1224 patients with 5621 phylogenetic trees across m=406 alterations.

For RECAP, in Christensen *et al.* 2020 the authors reported that the best performances are reached when only mutations appearing in at least 100 input patients are considered. This choice only arises from the empirical search of the best performances of RECAP on the considered dataset. From a theoretical perspective, instead, the removal of alterations from the input data reduces the amount of information that can be potentially exploited during training. So to have fair comparisons, we introduce a parameter *F* representing the minimum number of training patients where a mutation must appear so to be considered by RECAP and we compare our model and the other baselines with both the best version of RECAP (F=100) according to the authors and the standard version that considers all input mutations (F=0). We also consider RECAP with F=50 (Supplementary Material C.3, available as supplementary data at *Bioinformatics Advances* online), which has intermediate results (Fig. 9, available as supplementary data at *Bioinformatics Advances* online). This shows that the choice of *F* has a significant impact on the performances, and selecting the most appropriate value for *F* is difficult and time-consuming. The same experiment is repeated 10 times for each value of F∈{0,50,100}, where every repetition corresponds to a different dataset split into a training set with 80% of the patients and a test set with the remaining 20% of patients. For each repetition, the different methods are compared for three clustering sizes K∈{2,3,4}.

Figure 2 shows the performances of the methods, with our model CPhyT-GNN that outperforms all the baselines for every considered value of *K*. Note that the standard application of CloMu has considerably worse performances than all other methods, with an average percentage of test patients for which our model assigns a larger probability that is around 90%. This is an evidence that our EL strategy captures intra-tumor heterogeneity and is therefore beneficial for cancer progression modeling. Moreover, our model constantly outperforms the random baseline, highlighting that the embeddings computed by our model are meaningful. The baseline that directly clusters phylogenetic trees shows very low performances, mainly due to noise in the input trees. Indeed, the tree clustering baseline tends to output a very large cluster with almost all trees and K−1 clusters with only few trees, even when the filtering procedure detailed in Supplementary Material C.4, available as supplementary data at *Bioinformatics Advances* online is applied. This unveils the power of CPhyT-GNN in uncovering complex patterns in the input trees that consider also tree topology, in spite of noise. Oncotree2vec and the CloMu-based baseline have performances noticeably worse than those of our model, but both better than RECAP for every K∈{2,3,4}. Surprisingly, the performances of RECAP are constantly below those of our model, despite RECAP was originally thought for phylogenetic tree-based patient clustering, while our model is general-purpose.

#### 3.2.2 Acute myeloid leukemia

The dataset originally contains n=77 patients affected by Acute Myeloid Leukemia (Morita *et al.* 2020), with phylogenetic trees derived from the application of SCITE (Jahn *et al.* 2016) on high-throughput single-cell sequencing data using a panel of SNVs. After removing phylogenetic trees with more than ten edges as done by the authors of CloMu (Ivanovic and El-Kebir 2023), the dataset has n=75 patients with 105 trees over m=116 alterations.

Due to the high number of alterations with respect to the number of patients present in the dataset, as done by the authors of CloMu, we collapse alterations at gene level, leading to an analysis that considers m=22 mutated genes. By collapsing alterations at gene level, the initial topology of the input phylogenetic trees is preserved, but the same gene might mutate multiple times in the same phylogenetic tree, violating the infinite sites assumption. Our model CPhyT-GNN can still be applied in this situation while the other state-of-the-art methods, including RECAP, cannot. As a consequence, we could not include RECAP in the comparisons.

We run 10 times the same experiment with different random splits of the dataset into a training set with 70% of patients and a test set with the remaining 30% of patients. For each split, clusterings of sizes K∈{2,3,4} are considered. It is possible to see from Fig. 3 that our model outperforms all the baselines for all the values of K∈{2,3,4} on average. These results show that even for this dataset, where the number of patients is low, our method improves over currently available approaches.

### 3.3 Survival time analysis

As a second case study, we consider the application of the embeddings extracted using our model for survival analysis. For this task, the input is a dataset $D_{S}=\{(T_{i},(t_{i},\delta_{i})):i\in [n]\}$, where $T_{i}\in T$ is the phylogenetic tree describing tumor evolution in patient *i* while the pair $\left(t_{i},\delta_{i}\right)$ gives information about the survival time, as explained in Supplementary Material D.1, available as supplementary data at *Bioinformatics Advances* online.

The dataset that we consider contains patients affected by breast cancer and is created by considering the clinical data provided by Razavi et al. (2018) and the phylogenetic trees provided by the authors of RECAP (Christensen *et al.* 2020) using SPRUCE (El-Kebir *et al.* 2016). We pre-process the merged dataset by removing patients with missing data and patients with more than one phylogenetic tree, since the vast majority of patients has exactly one reconstructed phylogenetic tree. The final dataset has n=871 patients over m=379 SNVs. In the following sections, two types of experiments on the described dataset will be presented: a survival analysis of clusters computed based on the unsupervised embeddings provided by our model and the application of our model to directly predict survival times.

#### 3.3.1 Survival analysis of clusters

The aim is to assess whether clustering the embeddings computed by CPhyT-GNN is meaningful from a survival point of view. We train our model on the input phylogenetic trees, so to have an embedding for each patient in the dataset. To cluster patients, we apply the Lloyd’s algorithm (Lloyd 1982) with the *K*-means objective to the computed embeddings and values K∈{2,…,15}. Thus, we feed the Kaplan-Meier estimator with survival time data to find the survival distribution for the patients in each cluster within the same clustering.

Figure 4 shows the results of the described experiment with K=2; the results for other values of *K* are in Fig. 10, available as supplementary data at *Bioinformatics Advances* online. It is clearly visible that survival curves associated with different clusters within the same clustering have distinct behaviors. Furthermore, we apply the Log-Rank test to the estimated survival time distributions of different clusters in a clustering. The *P* values resulting from the application of the log-rank test to the computed clusterings are always very small ($<10^{-8}$), indicating that the clusterings produced using the embeddings extracted by our model are meaningful also from a survival point of view.

#### 3.3.2 Survival time prediction

We then evaluated whether our model can be used also to directly predict survival times of patients. The input dataset is split at random 10 times into a training set with 80% of patients and a test set with the remaining 20% of patients. We train our GNN-based model on the training set, in an unsupervised way, in order to have an embedding representing each patient based on the corresponding phylogenetic tree. Then, a Survival Support Vector Machine (Pölsterl et al. 2015) (SSVM) is trained using the training embeddings as feature vectors and the pairs $\left(t_{i},\delta_{i}\right)$ as labels. At inference time, an unseen phylogenetic tree is given as input to our trained model, which computes an embedding for it, fed then to the trained SSVM that outputs the predicted survival time. We also propose a supervised version of our model that simply has an additional linear head with a single output neuron on top of the original architecture. This slight modification allows us to train the model on the training set, using as input the phylogenetic trees and as labels the pairs $\left(t_{i},\delta_{i}\right)$.

To the best of our knowledge, no existing method in the literature predicts survival time from phylogenetic data. Note that Oncotree2vec cannot embed phylogenetic trees not included in the training process, making it impossible to embed test phylogenetic trees. Therefore, we train Oncotree2vec on both training and test phylogenetic trees, feeding the learnt embeddings to an SSVM trained only on the training embeddings and tested on the test ones. Note that in a real scenario in which we need to predict the survival time of an unseen phylogenetic tree *T*, Oncotree2vec has to be re-trained on the whole training set with the addition of *T*. This is a substantial difference with respect to CPhyT-GNN, which can just be fed with *T* and directly outputs the predicted embedding. Therefore, we also include CPhyT-GNN trained on both training and test phylogenetic trees, with training embeddings used to train an SSVM and test embeddings to test it, exactly as done for Oncotree2vec.

We also consider four baselines that use an SSVM to predict survival times given a vector (embedding), but using different methods to encode phylogenetic trees into vectors. First, we consider an *alteration encoding baseline*: given a phylogenetic tree *T*, it is encoded as a vector $s\in {\left\{0,1\right\}}^{m+2}$ with s[i]=1 if the label *i* appears in *T*. Note that the vector has m+2 components, because i=0 is used to represent the root and i=m+1 indicates the presence of unknown alterations. Second, we consider a *clone encoding baseline* that encodes *T* as a vector $t\in N^{m+2}$, where t[i] is the number of clones in which alteration *i* appears in *T*. Third, we consider the baseline that encodes *T* as the concatenation $u=s\|t\in N^{2(m+2)}$. The last baseline is obtained by computing a clustering with K∈N clusters of both training and test phylogenetic trees and then encodes each tree *T* as a one-hot vector $c\in {\left\{0,1\right\}}^{K}$ according to the cluster assigned to *T*. Supplementary Material D.2 and D.3, available as supplementary data at *Bioinformatics Advances* online, provide details on the four baseline encodings and on how the considered survival methods are trained, respectively.

**Figure 5:**
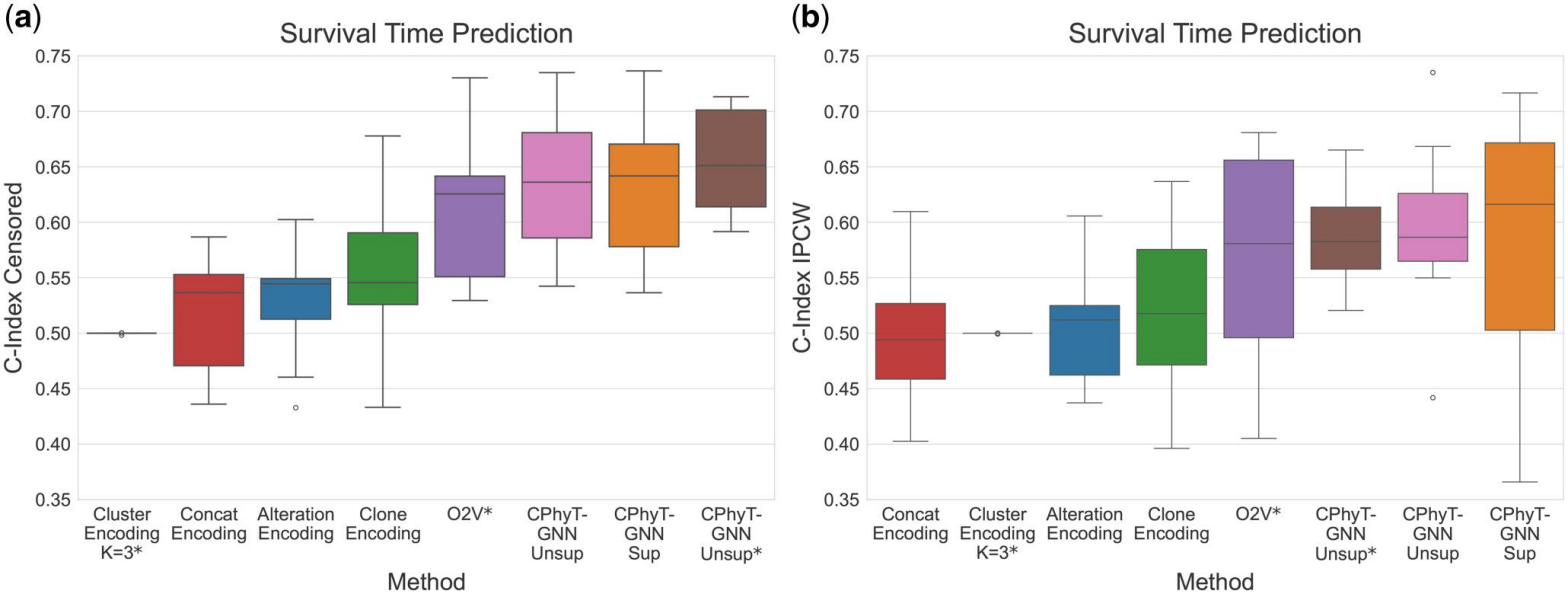
Comparison of different models for survival time prediction on the breast cancer dataset: considering (a) C-Index Censored and (b) C-Index IPCW. The symbol * identifies methods that learn embeddings using both training and test phylogenetic trees (but no access to survival variables); the other methods learn embeddings from training trees only.

Figure 5 reports the performances on test data of all considered methods according to two well-known metrics: the C-Index Censored (Harrell Jr *et al.* 1996) and the C-Index with Inverse Probability of Censoring Weighting (IPCW) (Uno *et al.* 2011). All the three versions of our model outperform all baselines, showing that taking into account also the topology of the input phylogenetic trees leads to significant improvements. Figure 11, available as supplementary data at *Bioinformatics Advances* online, shows that the performances of the cluster-based baseline do not improve if different values of *K* are considered. Oncotree2vec is the best of the baselines, but has performances worse than CPhyT-GNN even considering the unsupervised version of the latter that does not use test trees to learn embeddings. This confirms that the unsupervised embeddings computed with our model are general-purpose.

## 4 Discussion

We introduced CPhyT-GNN, a novel model to extract unsupervised general-purpose embeddings from phylogenetic trees. The model is based on a GNN, capable of extracting representations based not only on the alterations occurring in an input phylogenetic tree, but also on the topological relations among clones. We also proposed a method to encode the nodes of a phylogenetic tree that takes into account the complexity of coexisting clones in a phylogenetic tree. On simulated data, the results revealed that our model captures the topological relations among alterations in a phylogenetic tree. The experiments in two real world datasets showed that the unsupervised embeddings computed by our model are general-purpose and can be applied to different tasks. Indeed, the results showed that our model reaches state-of-the-art performances both on cancer progression modeling and survival time prediction, where we also proposed a supervised version of our model.

## Supplementary Material

vbag016_Supplementary_Data

## Data Availability

The data underlying this article are available in GitHub at https://github.com/VandinLab/CPhyT-GNN.
