# Supplementary material for: General-purpose topology-aware embedding of tumor phylogenetic trees with graph neural networks
Source: Bioinform Adv. 2026 Apr 26;6(1):vbag016. doi: 10.1093/bioadv/vbag016 (PMC13125753; doi:10.1093/bioadv/vbag016)
Supplement: vbag016_Supplementary_Data [file vbag016_supplementary_data.pdf]

General-Purpose Topology-Aware Embedding of Tumor Phylogenetic Trees with Graph Neural Networks

Supplementary Material

Paolo Bresolin
University of Padova, Italy

Fabio Vandin*
University of Padova, Italy

A Methods

A.1 Phylogenetic Tree Distance

As described in the main paper, our method CPhyT-GNN works with any tree distance function $d : \mathcal{T} \times \mathcal{T} \rightarrow \mathbb{R}^+$ suitable for the notion of phylogenetic tree that we propose. Among the distance functions present in the literature and commonly applied to phylogenetic trees [1, 2, 3], we decide to start from the *Ancestor-Descendant Distance* (ADD) [1] and adapt it to our definition of phylogenetic tree. In particular, we generalize it to node labels with multiple alterations.

Formally, let $T = (V, E)$ be a phylogenetic tree and let $u, v \in V$ be two nodes. If there exists a directed path from u to v in T, then u is said to be *ancestral* to v and, symmetrically, v is said to be *descendant* of u. We define as $A(T) = \{(u, v) : u \text{ is ancestral to } v \text{ in } T\}$ the set with all ancestor-descendant pairs in T. Since we have a set of alterations L_u labelling node u and a set of alterations L_v labelling node v, we define as $\phi(u, v) = \{(a, b) : a \in L_u,\ b \in L_v\}$ the set with all ancestor-descendant pairs of alterations between the two sets. Hence, the set with all ancestor-descendant pairs of alterations in T is defined as $\phi(T) = \bigcup_{(u,v)\in A(T)} \phi(u, v)$.

At this point, we define the revised ADD $d_{AD}(T_i, T_j)$ between a pair of phylogenetic trees T_i, $T_j \in \mathcal{T}$ as:

$$d_{AD}(T_i, T_j) = |\phi(T_i) \oplus \phi(T_j)|, \tag{S1}$$

where the symbol $\oplus$ denotes the symmetric set difference operation. In words, the revised ADD measures the number of ancestor-descentant pairs of alterations that are present in only one of the two input phylogenetic trees.

*Corresponding author: fabio.vandin@unipd.it

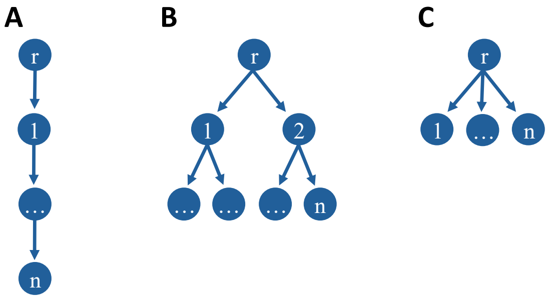

Figure S1: Base trees used to generate phylogenetic trees belonging to different clusters. The three trees all have the same alterations $\{1, \ldots, n\}$ as node labels, but different topologies. (**A**) Linear topology: each node, except for the leaf, has exactly one child, creating a chain of nodes. (**B**) binary topology: complete binary tree. (**B**) branching topology: the root is the only node that has children.

So to have a more precise distance measure, we add the artificial label *root* to the root node r of each phylogenetic tree T, such that its label set is $L_r = \{\text{root}\}$. This way, we are considering also the ancestor-descendant pairs $\phi(r, v)$ for every node v in T. Analogously, we set $L_v = \{\text{empty}\}$ for each node v that has no alteration label. Otherwise, nodes without alteration labels would not affect the ADD value and we would not exploit the topological information that they carry.

B Simulations

In this section, we describe how synthetic data is generated for each simulation and we discuss the results obtained by the application of our model. In every simulation, a synthetic dataset of phylogenetic trees is generated such that the trees are clustered into K groups based on some property. Then, our model is trained on all the generated phylogenetic trees so to compute unsupervised embeddings for them, which are fed to the Lloyd's algorithm [4] with the K-means objective, used to cluster the input data. The aim is to have a clustering that is as close as possible to the ground truth. To evaluate the performances of our model we consider the Rand Index [5] between the predicted clustering and the true one. Note that simulations I, II and III do not make use of the infinite sites assumption.

B.1 Simulation I

In simulation I, we create 3 base phylogenetic trees with $n = 8$ nodes, root excluded, such that they have the same alterations $A = \{1, \ldots, n\}$, but different topologies, as reported in Figure S1: branching, binary and linear. Starting from each base tree, we generate a cluster with 100 phylogentic trees. Let

B_i be a base tree and let C_i be the cluster that will contain all phylogenetic trees generated starting from B_i, $i \in \{1, 2, 3\}$. In detail, a phylogenetic tree $T_j^i = (V_j^i, E_j^i)$, $j \in \{1, \ldots, 100\}$ is initialized as a copy of the base tree B_i. Then, T_j^i is edited by applying to it t random operations, subsequently sampled uniformly at random from the following three:

- empty node insertion: a node v is sampled uniformly at random from V_j^i and an empty node is inserted as child of v;
- labelled node insertion: a node v' is created with as label an alteration sampled uniformly at random from $A \cup \{n+1, \ldots, 2n\}$. A node v is sampled uniformly at random from V_j^i and v' is inserted as child of v;
- node deletion: a node $v \in V_j^i \setminus \{r\}$ is sampled uniformly at random. If v is not a leaf, then its children are appended as children of the parent of v. Finally, v is removed from T_j^i.

This way trees within the same cluster have similar topologies while trees in different clusters have dissimilar topologies. Furthermore, it is clear that the generated phylogenetic trees cannot be clustered based only on the alterations that they carry.

We generate 10 random synthetic datasets for each value of $t \in \{1, \ldots, n\}$. Note that the binary topology can be considered as a trade-off between the linear and branching topologies and, especially when t approaches to n, it is very difficult to distinguish the trees belonging to the three different clusters. Therefore, we consider also a simplified version of the simulation, named simulation Ib, where there are only $K = 2$ clusters with linear and branching topologies, without considering the binary base tree. Instead, we call simulation Ia the standard version of the simulation, with $K = 3$ and all the three kinds of topologies.

Despite being very difficult, Figure S2 shows that the Rand Index is very high also when all the three topologies are considered. As expected, the performances decrease as t increases, since the large number of applied random changes potentially disrupts the initial topology of the base trees. However, the Rand Index is steadily above 0.7 $\forall t \in \{1, \ldots, 8\}$ and around 0.9 when $t < 6$. Finally, as conjectured, the application of our method to simulation Ib provides better performances, with the Rand Index that is constantly above 0.8 for all the tested values of t.

B.2 Simulation II

In simulation II we consider the same tree topologies used in simulation I, but this time we create random trees by randomly assigning alterations to the nodes of the base trees. We create $K = 3$ clusters C_1, C_2, C_3, where each cluster contains 100 trees all with the same topology, but random alterations. A random phylogenetic tree T_j^i, $i \in \{1, \ldots, K\}$, $j \in \{1, \ldots, 100\}$ is generated such that it has n nodes, the topology related to cluster C_i and every node labelled by an alteration chosen uniformly at random from the set $A = \{1, \ldots, Kn\}$.

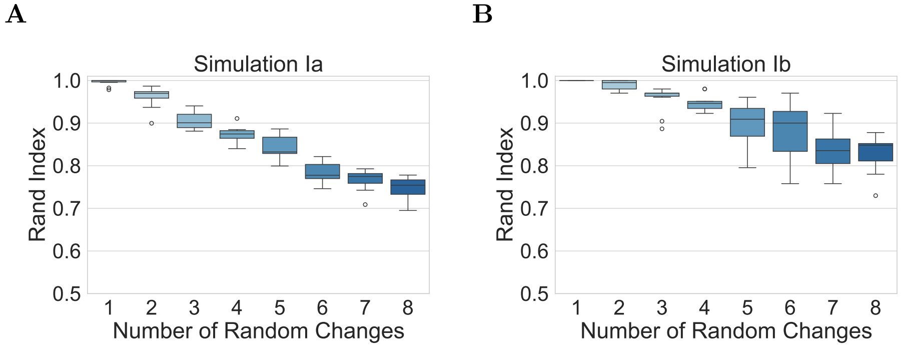

Figure S2: Results obtained from the application of our method to simulation I. In both plots, the x-axis represents the number of random changes $t \in \{1, \ldots, n\}$ applied to a base tree B_i to generate each tree T_j^i, $i \in \{1, \ldots, n\}$, $j \in \{1, \ldots, 100\}$. The y-axis reports the Rand Index between the clustering computed applying the Lloyd's algorithm to the unsupervised embeddings computed by our model and the ground truth clustering. Each box plot is created based on 10 repetitions of the same experiment with different randomly generated synthetic datasets. The black line inside the box plots stands for the median value across all 10 repetitions while circles represent outliers. (**A**) Simulation Ia: all 3 topologies are considered: linear, binary and branching. (**B**) Simulation Ib: 2 topologies are considered: linear and branching.

Again, we consider two versions of the simulation: simulation IIa with all three topologies and simulation IIb that considers only the linear and branching topologies. The results of the application of our model to both simulations across 10 randomly generated datasets and different number of nodes $n \in \{8, \ldots, 20\}$ are shown in Figure S3.

In simulation IIa the Rand Index is almost always above 0.9, with only few exceptions. In particular, we observe that the performances tend to increase as n increases, with the best Rand Index for $n = 20$ and the worst score when $n = 8$. The explanation probably relies on the dependence of the size of the alteration set $A_a = \{1, \ldots, 3n\}$ from n. Indeed, a larger alteration set reduces the probability of having common repeated patterns of relations among alterations in random trees with different topologies.

In simulation IIb, instead, the performances approach a Random Index of 1 when $n < 14$ and tends to decrease as the number of nodes increases. This behavior is due to a smaller alteration set $A_b = \{1, \ldots, 2n\}$ with respect to the alteration set $A_a = \{1, \ldots, 3n\}$ used in simulation IIa, which induces a larger probability of phylogenetic trees with repeated random patterns. Furthermore, due to the definition of ADD, the distance between two phylogenetic trees with the same linear topology, but random alterations as node labels, is considerably large and makes very difficult for our model to consider the two trees as belonging to the same cluster.

A **B**

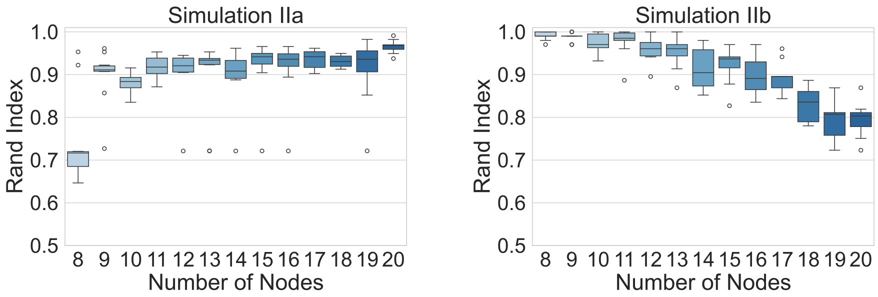

Figure S3: Results obtained from the application of our method to simulation II. The box plots are created from the repetition of the simulations with 10 different randomly generated datasets. The median value is highlighted in each box plot by means of a black line, while the circles represent outliers. The simulations are repeated for different sizes of the generated phylogenetic trees, reported in the x-axis. The y-axis shows the Rand Index between the clutstering predicted using our model and the true one. (**A**) Simulation with $K = 3$ clusters, each of them with trees with a different topology: linear, branching or binary. (**B**) Simulation with $K = 2$ cluster with phylogenetic trees that have linear or branching topology.

In any case, the performances of our model are meaningfully high even in this very complex simulation, regardless of the types of considered topologies.

B.3 Simulation III

Since we want to assess the capability of our GNN-based model to capture the topology of the input phylogenetic trees, we perform a simulation with all trees that are unlabelled. Therefore, the only information to distinguish them relies in their topology. We consider the branching and linear topologies already defined in the previous sections and we create $K = 2$ clusters of phylogenetic trees such that trees with the same topology are placed in the same cluster. Specifically, we set a maximum number $n_{\max}$ of nodes, root excluded, and we generate all the trees with $n \in \{1, \ldots, n_{\max}\}$ empty nodes and one of the two considered topologies. By construction, the two clusters both contain $n_{\max}$ phylogenetic trees.

Since there is no randomness in the data generation for this simulation, there is no need to perform the same simulation multiple times. Figure S4 shows the performances of our model on simulation III for different values of $n_{\max} \in \{8, \ldots, 20\}$. The performances are noticeable, with a Rand Index that is always around 0.9 and often above it. The fact that there is an improvement as $n_{\max}$ increases is related to the increasing number of samples present in the dataset, that allows our GNN-based model to better capture the dissimilarity

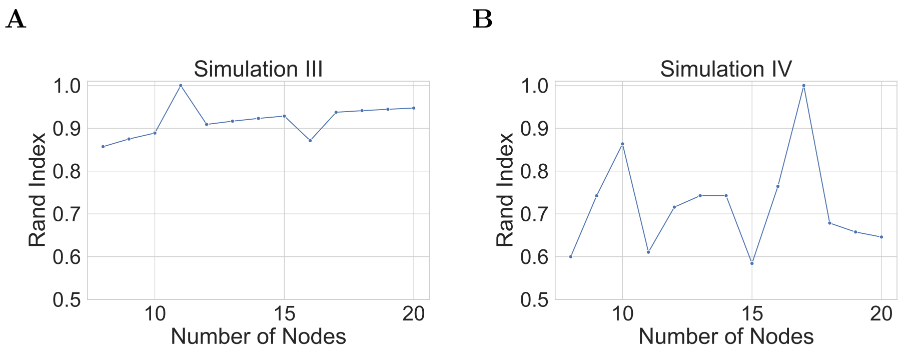

Figure S4: Results obtained from the application of our method to simulations III and IV. (**A**) Simulation III: the plot shows the Rand Index between the clustering predicted by our model and the ground truth, reported in the y-axis, across different maximum number of non-root nodes in the generated trees. (**B**) Simulation IV: the y-axis represents the Rand Index between the true clustering and the one predicted by our method. The x-axis reports the tested sizes of the generated phylogenetic trees in terms of number of non-root nodes.

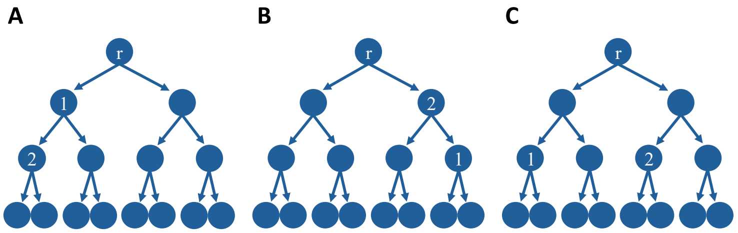

Figure S5: Different types of relations between the two alterations 1 and 2 considered in simulation IV. The generated dataset has $K = 3$ clusters of phylogenetic trees such that all trees within a cluster have the same type of relation between alterations 1 and 2. (**A**) Alteration 1 is ancestral to 2, meaning that it has been acquired before 2. (**B**) Alteration 1 is descendant of 2, that is, it has been acquired after 2. (**C**) Alterations 1 and 2 are mutually exclusive, i.e., there is no path from the root to any other node of the phylogenetic tree in which both 1 and 2 appear.

between the two considered topologies.

B.4 Simulation IV

With the previous simulations, we tested the capability of our model to distinguish phylogenetic trees with distinct topologies. Now we test whether our method also captures topological relations among alterations specific to tumor phylogenetic trees.

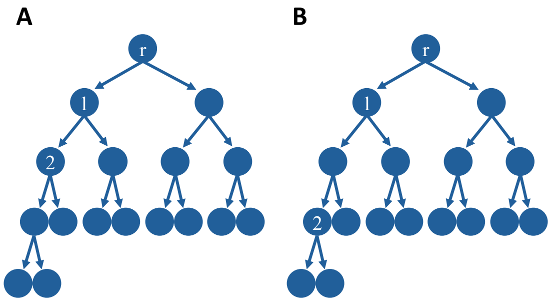

Figure S6: Two phylogenetic trees with the same number $n = 16$ of non-root nodes, the same binary topology and the same type of relation between alterations 1 and 2. Alteration 1 labels the same node in both trees, while alteration 2 is placed at different depths. Despite the type of relation between 1 and 2 is the same in both trees, the fact that 2 is farther from 1 induces different ancestry sets. Since the two phylogenetic trees have different ancestry sets, the distances between them is different from 0. (**A**) The ancestry set is $A_a = \{(\text{root}, 1), (\text{root}, 2), (\text{root}, \text{empty}), (1, 2), (1, \text{empty}), (2, \text{empty}), (\text{empty}, \text{empty})\}$. (**B**) The ancestry set is $A_b = \{(\text{root}, 1), (\text{root}, 2), (\text{root}, \text{empty}), (1, 2), (1, \text{empty}), (2, \text{empty}), (\text{empty}, \text{empty}), (\text{empty}, 2)\}$.

We define two alterations $1, 2$ and we consider the following three possible relations among them:

- 1 is ancestral to 2, that is, 1 appears before 2 in the path from the root to 2;
- 1 is descendant of 2, i.e., there exists a path from 2 to a leaf that touches the node with label 1;
- 1 and 2 are mutually exclusive, meaning that there is no path that contains both 1 and 2.

For each type of relation between 1 and 2 described above, we create a cluster with all possible phylogenetic trees with n nodes and the two alterations placed in different nodes. All other nodes are left empty. Figure S5 provides as example one phylogenetic tree for each cluster when $n = 14$.

Since the revised ADD is sensitive to all relations among all labels in the tree, we do not allow the alterations 1 and 2 to label leaf nodes, still allowing for trees with the same type of relation, but different ancestry sets, as reported in Figure S6. Note that the generated clusters are unbalanced, because the number of all possible mutually exclusive relations between 1 and 2 is significantly larger than the number of all possible relations with 1 as ancestor of 2 or viceversa.

Figure S4 witnesses that, despite the complexity of the problem, our model is capable of producing a clustering that is not far from the ground truth. We consider $n \in \{8, 20\}$ and with $n = 17$ we observe the best Rand Index value of

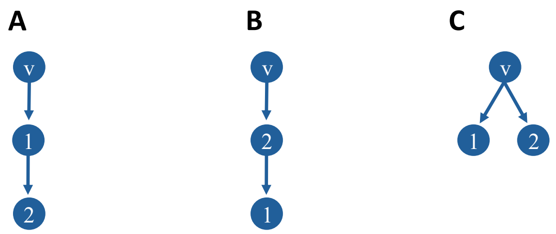

Figure S7: Subtrees appended to the phylogenetic trees generated in simulation V. Each subtree is present only in the trees of the corresponding cluster. Let $T = (V, E)$ be the base phylogenetic tree with n non-root unlabelled nodes. Given $v \in V$, a tree $T' = (V', E')$ is generated starting from T by appending to v one of the three structures as represented in the figure. (**A**) The inserted subtree introduces a parent-child relation between 1 and 2, resulting in a tree $T' = (V', E')$ such that $V' = V \cup \{1, 2\}$ and $E' = E \cup \{(v, 1), (1, 2)\}$ (**B**) The inserted subtree introduces a parent-child relation between 2 and 1, resulting in a tree $T' = (V', E')$ such that $V' = V \cup \{1, 2\}$ and $E' = E \cup \{(v, 2), (2, 1)\}$. (**C**) The inserted subtree introduces a relation of mutual exclusivity between 1 and 2, resulting in a tree $T' = (V', E')$ such that $V' = V \cup \{1, 2\}$ and $E' = E \cup \{(v, 1), (v, 2)\}$.

1.0, coinciding with a perfect clustering of the input trees. Finally, this time there is not a clear trend of the Rand Index based on n.

B.5 Simulation V

To further inquire the power of our model in uncovering topological relations between two alterations that are typical to real tumor phylogenetic tree, we create simulation V. This time the base tree $T_b = (V_b, E_b)$ is the same for all clusters and consists in a binary phylogenetic tree with n unlabelled nodes. We consider the same three relations between two alterations 1 and 2 described in the previous section. However, this time we do not directly insert them in the base tree. Instead, given a node $v \in V_b$, we add a subtree rooted in v with a specific relation between 1 and 2, as shown in Figure S7. Since the base phylogenetic tree T_b has n nodes, we generate n trees for each substructure by appending it every time to a different node $v \in V_b$.

By construction, this time the distance between two phylogenetic trees that have the same substructure rooted at different levels is the same, because the distance between 1 and 2 is always preserved. As a consequence, we expect our model to have better performances with respect to simulation IV. In particular, to have a more detailed evaluation of our model on this simulated scenario, we consider three versions of the simulation:

- simulation Va: all the three types of relations are considered, with the result of $K = 3$ clusters;

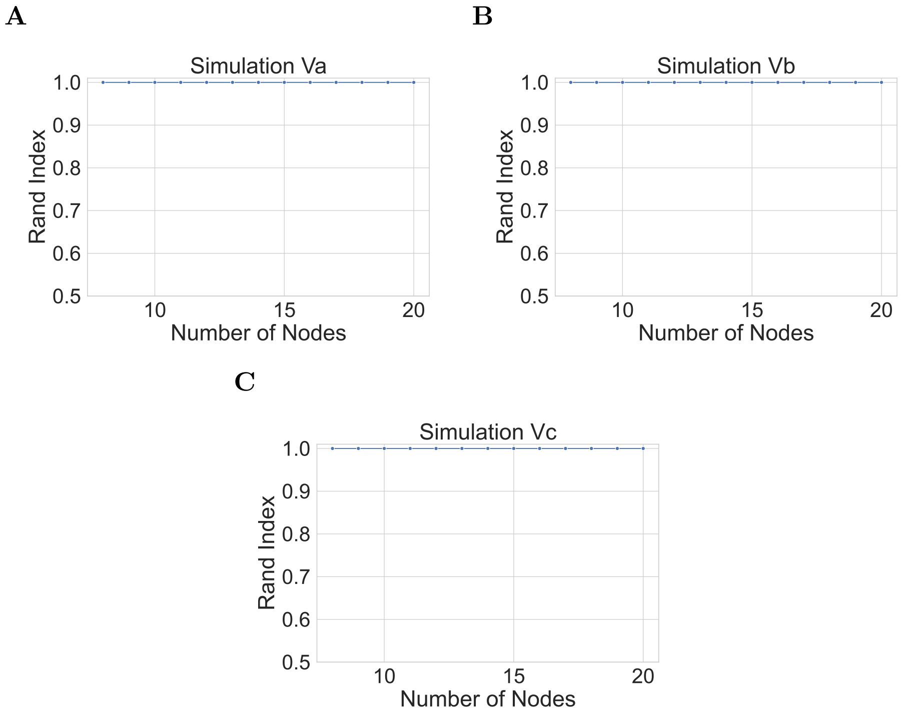

Figure S8: Results obtained from the application of our method to simulation V. In every plot, the Rand Index across different number of nodes n of the generated trees is represented. (**A**) $K = 3$ clusters of trees with different relations: 1 ancestral to 2, 2 ancestral to 1 and $1, 2$ mutually exclusive. (**B**) $K = 2$ clusters of trees with different relations: 1 ancestral to 2 and mutual exclusivity between 1 and 2. (**C**) $K = 2$ clusters of trees with different relations: 1 ancestral to 2 and 2 ancestral to 1.

- simulation Vb: only the relations 1 ancestral to 2 and $1, 2$ mutually exclusive are considered, producing $K = 2$ clusters;
- simulation Vc: only the opposite relations 1 ancestral to 2 and 2 ancestral to 1 are considered, creating $K = 2$ clusters.

Figure S8 reports the results for all the three types of simulations across different values of $n \in \{8, \ldots, 20\}$. As conjectured, the performances of our model are better in this simulation than simulaton IV. In addition, the Rand Index is 1.0 in every simulation and for every tested value of n. This is a clear evidence that our model, thanks to the GNN at its basis, can distinguish phylogenetic trees with distinct topological relations specific to real tumor data.

C Cancer Progression Modeling

C.1 CloMu-based Clustering Method

In this section, we provide a detailed explanation of the CloMu-based baseline, used for comparisons in the cancer progression modeling task. We consider the general case in which the input consists of a multiset $\mathcal{I} = \{\mathcal{T}_i : i \in [n]\}$, where $\mathcal{T}_i = \{T^i_\ell : \ell \in [|\mathcal{T}_i|]\}$ is the set of plausible trees for patient $i \in [n]$. The objective is to find a clustering of size $K > 1$ of the n input patients, using as data the multiset of phylogenetic trees $\mathcal{I}$.

The algorithm takes as input the clustering $\mathcal{C} = \{C_1, \ldots, C_K\}$ obtained by the random clustering baseline explained in the main text. Just to recap, the baseline randomly clusters the patients in $\mathcal{I}$ such that the output clusters contain the same number of patients present in the clusters computed by CPhyT-GNN. At this point, a different CloMu instance M_j is trained on each cluster C_j, $j \in [K]$. Then, every patient $i \in [n]$ is assigned to the cluster $C^*_j = \arg\min_{j \in [K]} 1/|\mathcal{T}_i| \sum_{T^i_\ell} M_j(T^i_\ell)$, where $M_j(T^i_\ell)$ is the probability assigned by M_j to T^i_ℓ. In words, we are re-assigning patients to the clusters of phylogenetic trees that have the closest distribution according to train CloMu models.

C.2 Patients with Multiple Phylogenetic Trees

Two real world datasets are used for testing our method on the cancer progression modeling task: one with patients affected by acute myeloid leukemia (AML) [6] and the other with patients affected by breast cancer [7]. Since such datasets were used also by the authors of CloMu, we directly consider as input the phylogenetic trees they provide. However, in both datasets there are patients with more than one plausible phylogenetic tree due to uncertainty when inferring them from sequencing data. Therefore, for each patient included in the training set, we select uniformly at random a single phylogenetic tree to be used as input to our model and to Oncotree2vec [8], designed to work directly on trees and not on patients. The CloMu-based clustering method and RECAP [9], instead, take as input all phylogenetic trees for each patient, still providing a clustering of patients, because they are thought to allow for patients with multiple trees.

At inference time, when the different methods need to be evaluated on an unseen test set, all phylogenetic trees are considered, allowing for patients with more than one plausible tree regardless of the method to be evaluated. Indeed, the different models are not applied to test data, which is fed only to multiple CloMu instances. Given a patient $i \in [n]$ with a set $\mathcal{T}_i = \{T^i_j : j \in [|\mathcal{T}_i|]\}$ of plausible trees, a CloMu instance predicts a set of probabilities $\mathcal{P}_i = \{P^i_j : j \in [|\mathcal{T}_i|]\}$, where P^i_j is the probability of tree T^i_j. We compute the probability $P^i = \frac{1}{|\mathcal{T}_i|} \sum_{j=1}^{|\mathcal{T}_i|} P^i_j$ of patient i as the average of the probabilities of the corresponding trees. Therefore, we evaluate the different methods on unseen test data by comparing the probabilities assigned to patients, allowing for patients

with multiple phylogenetic trees.

C.3 RECAP Parameter F

In the main text, we explained that to run RECAP [9] it is necessary to provide as input the minimum number $F \in \mathbb{N}$ of training patients where an alteration must appear so to be considered by the model. F is determined experimentally, ending up with $F = 100$ as the best value for the breast cancer dataset [7]. However, reducing the set of alterations considered by the model, despite simplifying the task, loses some information that can potentially be useful to cluster the input patients. Indeed, both inter and intra-tumor heterogeneity are mitigated by the introduction of F. CPhyT-GNN, on the other hand, always considers all the alterations present in the training set, exploiting the full potential of the input data.

Notice that the selection of the most appropriate value for the parameter F is highly time-consuming, because it requires to train and evaluate the entire model for every assignment of F and for every considered dataset. In addition, the choice of F is very important, since it noticeably affects the performances of RECAP, as shown by Figure S9.

C.4 Filtering for Clustering

With the objective of improving clustering performances, we pre-process samples before clustering with CPhyT-GNN, Oncotree2Vec and the tree clustering baseline. In what follows, we will explain the adopted pre-processing scheme, showing how it adapts to each different clustering method.

In general, let $X = \{x_1, \dots, x_n\} \subset \mathbb{R}^\ell$ be the set of samples to be clustered and let $d : X \times X \to \mathbb{R}^+$ be the distance metric used to cluster X. We compute the mean point $\bar{x} = \frac{1}{n}\sum_{i=1}^{n} x_i$ and the average distance $d(\bar{x}, X) = \frac{1}{n}\sum_{i=1}^{n} d(\bar{x}, x_i)$ from the points in X to the mean point $\bar{x}$. Then, we create the subset $S = \{x_i \in X : d(\bar{x}, x_i) \leq \gamma d(\bar{x}, X)\} \subseteq X$ of points at distance at most γ times the average distance $d(\bar{x}, X)$ from the mean point $\bar{x}$, where $\gamma \in \mathbb{R}$. In practice, S contains the points in a ball centered in the mean point $\bar{x}$ of radius $\gamma d(\bar{x}, X)$. The clustering algorithm is then applied only to the points in S using the distance function d, producing a clustering $\mathcal{C} = \{C_1, \dots, C_K\}$, where $K \in \mathbb{Z}^+$ is the number of clusters. Finally, every point $x \in X \setminus S$ is assigned to the closest cluster $C_{j^*} = \arg\min_{j \in \{1,\dots,K\}} d(x, C_j)$, where $d(x, C_j) = 1/|C_j| \sum_{x_\ell \in C_j} d(x, x_\ell)$.

The filtering procedure explained above is directly applied to the embeddings output from CPhyT-GNN using the Euclidean distance as function d and to the embeddings computed by Oncotree2vec with d being the cosine distance. Indeed, the embeddings learnt by CPhyT-GNN are clustered using the Lloyd's algorithm in the K-means framework, while hierarchical clustering with cosine distance is used to cluster the embeddings from Oncotree2vec.

The filtering algorithm is slightly modified to the case of the tree clustering baseline, since in this case we do not have points in $\mathbb{R}^\ell$ to be clustered, but phy-

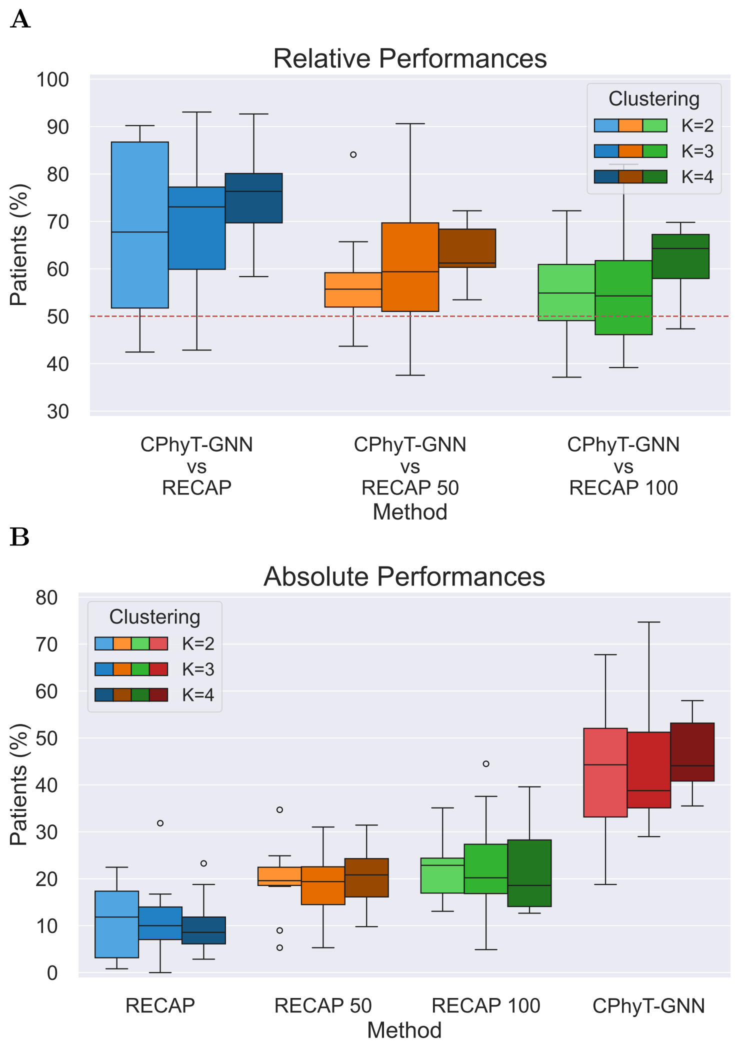

Figure S9: Comparison of CPhyT-GNN and RECAP with different values of $F \in \{0, 50, 100\}$ on the cancer progression task. F indicates the minimum number of occurrences of an alteration not to be discarded by RECAP. The choice of F has a noticeable impact on RECAP's performances. In the plots, RECAP refers to the standard application of RECAP with $F = 0$. Each box plot corresponds to 10 experiment repetitions. Colors of different brightness encode different values of $K \in \{2, 3, 4\}$. The line in each box plot represents the median value and the empty circles are outliers. (**A**) Percentage of test patients whom CPhyT-GNN assigns a larger score than all other RECAP versions. (**B**) Percentage of test patients whom each method assigns a score larger than all other models.

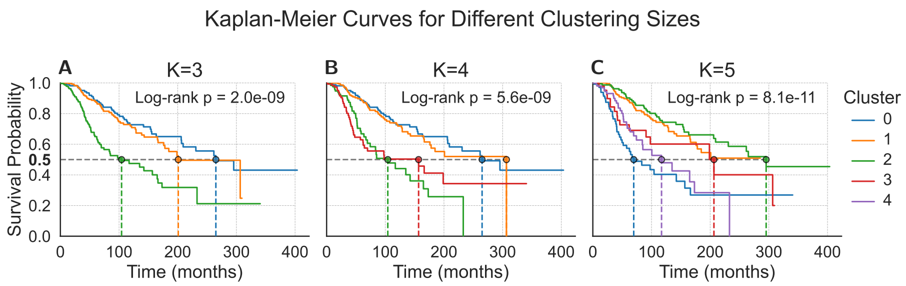

Figure S10: Survival time distributions for different clusters of clusterings with $K \in \{3, 4, 5\}$ computed by CPhyT-GNN on the breast cancer dataset. Different colors encode distinct clusters. The p-value from the Log-Rank test is reported for every clustering.

logenetic trees. Specifically, consider the input set $\mathcal{T} = \{T_1, \ldots, T_n\}$ of phylogenetic trees to be clustered by means of a tree distance function $d_T : \mathcal{T} \times \mathcal{T} \rightarrow \mathbb{R}^+$, which is the *Ancestor-Descendant Distance* in our experiments. The mean distance $\bar{d}_T = 1/\binom{n}{2} \sum_{i=1}^{n} \sum_{j=i+1}^{n} d_T(T_i, T_j)$ among all trees is computed. We also compute the mean distance $d_T(T_i, \mathcal{T}) = \frac{1}{n-1} \sum_{j \neq i} d_T(T_i, T_j)$ from each phylogenetic tree $T_i \in \mathcal{T}$ to all the other trees in $\mathcal{T}$. At this point, only the phylogenetic trees in the subset $\mathcal{S} = \{T_i \in \mathcal{T} : d_T(T_i, \mathcal{T}) \leq \gamma \bar{d}_T\} \subseteq \mathcal{T}$ are fed to the clustering algorithm, which is hierarchical clustering with d_T as distance metric. As before, given the computed clustering $\mathcal{C} = \{C_1, \ldots, C_K\}$, every phylogenetic tree $T \in \mathcal{T} \setminus \mathcal{S}$ is assigned to the closest cluster $C_{j^*} = \arg\min_{j \in \{1, \ldots, K\}} d_T(T, C_j)$, where $d_T(T, C_j) = 1/|C_j| \sum_{T_\ell \in C_j} d_T(T, T_\ell)$.

D Survival Time

D.1 Data

Survival time data is subjected to *right censoring*, a condition that makes survival time only partially known for some patients, due to patients leaving the study before it ends or who die after the end of the study. We define as $t_i > 0$ the observed time for patient $i \in [n]$ and $\delta_i \in \{0, 1\}$ the event indicator function, taking value $\delta_i = 1$ if death was observed for patient i and value $\delta_i = 0$ in case of censoring.

Therefore, for the survival analysis of clusters and the survival time prediction task that we consider in the experiments, the input consists of a dataset $D_S = \{(T_i, (t_i, \delta_i)) : i \in [n]\}$, where $T_i \in \mathcal{T}$ is the phylogenetic tree describing tumor evolution in patient i while the pair (t_i, δ_i) gives information about the survival time.

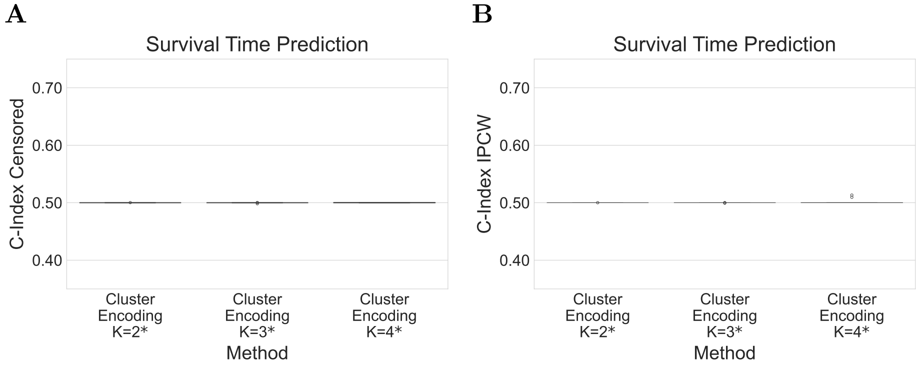

Figure S11: Comparison of the survival prediction methods based on the cluster encoding of patients with $K \in \{2, 3, 4\}$ on the breast cancer dataset, considering the C-Index Censored (**A**) and the C-Index IPCW (**B**). As it is possible to see, the cluster encoding baseline performs very poorly regardless of the clustering size K.

D.2 Survival Time Prediction Baselines

To the best of our knowledge, in the literature there is no model that takes as input a phylogenetic tree and predict survival time for the corresponding patient as output. We define four different baselines for the comparisons with Oncotree2Vec and the three versions of CPhyT-GNN that we propose.

Let $D_S = \{(T_i, (t_i, \delta_i)) : i \in [n]\}$ be the training data available for the survival prediction task and let $[m] = \{1, \dots, m\}$ be the set of alterations appearing in the phylogenetic trees in D_S. The *alteration encoding* baseline encodes each phylogenetic tree T_i as a vector $\mathbf{s}_i \in \{0, 1\}^{m+2}$, where each component $\mathbf{s}_i[j] = 1$ if alteration $j \in [m]$ is present in T_i and $\mathbf{s}_i[j] = 0$ otherwise. Component $\mathbf{s}_i[0]$ denotes the presence of the root in the input phylogenetic tree, while the last component $\mathbf{s}_i[m + 1] = 1$ if the phylogenetic tree contains unknown alterations.

The *clone encoding* baseline works very similarly to the previous one, encoding each phylogenetic tree T_i as a vector $\mathbf{t}_i \in \mathbb{N}^{m+2}$, where $\mathbf{t}_i[j]$ is the number of clones in T_i in which alteration $j \in [m]$ is present. Remind that, given a phylogenetic tree $T_i = (V_i, E_i)$, every node $v_i \in V_i$ induces a clone $c(v_i) = \bigcup_{u_i \in \Pi_{r_i, v_i}} L_{u_i}$, where Π_{r_i, v_i} being the set of nodes in the path from the root $r_i \in V_i$ to v_i. Analogously to the alteration encoding baseline, $\mathbf{t}_i[0]$ denotes the presence of the root in the input phylogenetic tree, while the last component $\mathbf{s}_i[m + 1]$ is set to the number of clones in T_i with unknown alterations. The *concatenation encoding* baseline simply maps each phylogenetic tree T_i to the vector $\mathbf{u}_i = \mathbf{s}_i \parallel \mathbf{t}_i \in \mathbb{N}^{2(m+2)}$.

Let $U_S = \{(T_\ell, (t_\ell, \delta_\ell)) : \ell \in [n_U]\}$ be the test data for the survival prediction task, consisting of n_U total samples. The *cluster encoding* baseline, exactly as Oncotree2Vec, is fitted on the entire dataset $D_S \cup U_S$. Specifically, the phylogenetic trees in $D_S \cup U_S$ are clustered into $K \in \mathbb{N}$ clusters and every

phylogenetic tree T is encoded as a one-hot vector $\mathbf{c} \in \{0,1\}^K$ such that $\mathbf{c}[j] = 1$ if T is assigned to cluster $j \in \{1, \ldots, K\}$ and all other components are set to 0.

Given the input training set $D_S = \{(T_i, (t_i, \delta_i)) : i \in [n]\}$, T_i is fed to whatever considered encoding function $e(\cdot)$ (either one of the four baselines described above or Oncotree2Vec or CPhyT-GNN), producing as output an encoding $e(T_i)$ of T_i for all patients $i \in [n]$. Then, an SSVM is trained end-to-end using the training data $e(D_S) = \{(e(T_i), (t_i, \delta_i)) : i \in [n]\}$ so to predict survival times.

D.3 Survival Time Prediction Loss Functions

Let $D_S = \{(T_i, (t_i, \delta_i)) : i \in [n]\}$ be the input training set for the task of survival time prediction. In the experiments related to this task, we consider many distinct methods that predict survival times from an input set of phylogenetic trees. As explained in section D.2, all the methods, except for the supervised version of CPhyT-GNN, are composed by an encoding function that maps phylogenetic trees into vectors and an SSVM trained on the training encodings and evaluated on the test ones. In what follows, we explain how the SSVMs are trained and how the supervised version of CPhyT-GNN is optimized.

Define as $\mathcal{P} = \{(i,j) : t_i > t_j \wedge \delta_j = 1\}_{i,j \in [n]}$ the set of *comparable pairs* and let $\mathbf{x}_i, \mathbf{x}_j$ be the two feature vectors, computed using whatever phylogenetic tree encoding method, of two phylogenetic trees T_i, T_j such that $(i,j) \in \mathcal{P}$. The loss function between $\mathbf{x}_i$ and $\mathbf{x}_j$, used to train an SSVM, is defined as:

$$\mathcal{L}(\mathbf{x}_i, \mathbf{x}_j) = \frac{1}{2}\mathbf{w}^\top \mathbf{w} + \frac{\alpha}{2} \sum_{(i,j) \in \mathcal{P}} \max(0, 1 - (\mathbf{w}^\top \mathbf{x}_i - \mathbf{w}^\top \mathbf{x}_j))^2, \tag{S2}$$

where $\mathbf{w}$ is the vector parameters learnable by the SSVM and α is a hyper parameter used to balance the real contribution of the loss with the regularization term $\frac{1}{2}\mathbf{w}^\top \mathbf{w}$. Note that Equation S2 addresses the prediction problem as a ranking problem rather than a regression task, as commonly done in the literature, due to the hardness of predicting survival times. The idea is that we want the predicted survival times to have a ranking that matches the ground truth ranking among true survival times as much as possible.

So to have the fairest comparisons possible between the supervised version of CPhyT-GNN and the other survival prediction methods, we train it with exactly the same loss function used to train the SSVMs on top of the other models, removing the term related to regularization since it is already applied inside the optimizer used for training:

$$\mathcal{L}(T_i, T_j) = \sum_{(i,j) \in \mathcal{P}} \max(0, 1 - (g_\theta(T_i) - g_\theta(T_j))^2, \tag{S3}$$

where $g_\theta(T_i)$ is the output of the model with input T_i and θ are the parameters of the model.

References

[1] Kiya Govek, Camden Sikes, Yangqiaoyu Zhou, and Layla Oesper. GraPhyC: Using consensus to infer tumor evolution. *IEEE/ACM Trans. Comput. Biol. Bioinform.*, 19(1):465–478, January 2022.

[2] Zach DiNardo, Kiran Tomlinson, Anna Ritz, and Layla Oesper. Distance measures for tumor evolutionary trees. *Bioinformatics*, 36(7):2090–2097, 11 2019.

[3] Hamed Vasei, Mohammad-Hadi Foroughmand-Araabi, and Amir Daneshgar. Weighted centroid trees: a general approach to summarize phylogenies in single-labeled tumor mutation tree inference. *Bioinformatics*, 40(7):btae120, 2024.

[4] Stuart Lloyd. Least squares quantization in pcm. *IEEE transactions on information theory*, 28(2):129–137, 1982.

[5] William M Rand. Objective criteria for the evaluation of clustering methods. *Journal of the American Statistical association*, 66(336):846–850, 1971.

[6] Kiyomi Morita, Feng Wang, Katharina Jahn, Tianyuan Hu, Tomoyuki Tanaka, Yuya Sasaki, Jack Kuipers, Sanam Loghavi, Sa A Wang, Yuanqing Yan, et al. Clonal evolution of acute myeloid leukemia revealed by high-throughput single-cell genomics. *Nature communications*, 11(1):5327, 2020.

[7] Pedram Razavi, Matthew T Chang, Guotai Xu, Chaitanya Bandlamudi, Dara S Ross, Neil Vasan, Yanyan Cai, Craig M Bielski, Mark TA Donoghue, Philip Jonsson, et al. The genomic landscape of endocrine-resistant advanced breast cancers. *Cancer cell*, 34(3):427–438, 2018.

[8] Monica-Andreea Baciu-Drăgan and Niko Beerenwinkel. Oncotree2vec—a method for embedding and clustering of tumor mutation trees. *Bioinformatics*, 40(Supplement_1):i180–i188, 2024.

[9] Sarah Christensen, Juho Kim, Nicholas Chia, Oluwasanmi Koyejo, and Mohammed El-Kebir. Detecting evolutionary patterns of cancers using consensus trees. *Bioinformatics*, 36(Supplement_2):i684–i691, 2020.
